# Comparative analysis of physical, morphological, tensile and thermal stability characteristics of raw and alkali treated novel *Tinospora cordifolia* natural fiber

**DOI:** 10.1038/s41598-025-03627-y

**Published:** 2025-05-28

**Authors:** Jamaluddin Hindi, K. Muralishwara, B. M. Gurumurthy

**Affiliations:** Department of Mechanical & Industrial Engineering, Manipal Institute of Technology, Manipal Academy of Higher Education, Manipal, India

**Keywords:** *Tinospora cordifolia* fiber, Crystallinity, Functional groups, Surface morphology, Single fiber tensile test, Thermal stability, Engineering, Materials science

## Abstract

This research paper presents comparative analysis of morphological, physical, tensile and thermal properties of raw and NaOH treated natural fiber extracted from the stems of *Tinospora cordifolia* herbaceous vine. Diameter & density measurements, X-ray Diffraction (XRD) analysis, Scanning Electron Microscopy (SEM), Fourier Transform Infrared (FTIR) spectroscopy, single fiber tensile tests and Thermogravimetric analysis (TGA) were carried out. The diameter reduced and density increased post treatment. SEM images revealed the removal of non-cellulosic contents, finer fibrillation, more clear hexagonal structure and formation of pores & voids post treatment. The Crystalline Index (C.I) and Crystallite Size (C.S) obtained from the XRD spectra enhanced after treatment which is evident from the absence of amorphous shoulders/bumps. FTIR analysis have revealed the removal of constituents such as hemicellulose, lignin and wax. The tensile strength enhanced, Young’s modulus and percent elongation at break reduced post treatment. The thermal stability enhanced; however, the maximum degradation showed a slight reduction.

## Introduction

During the last ten years, much attention has been paid to natural fibers as reinforcement materials in polymer composites due to their environmental benefits, renewability, and availability^1–4^. Natural fibers have several desirable properties: low weight, biodegradability, high specific strength and stiffness, and relatively low density compared with synthetic glass or carbon fibers^4–6^. Advantages arising thereof make them suitable for a wide range of applications like automotive components, construction materials, packaging, and consumer goods^7^. In recent years the growing emphasis on sustainability and the decline of non-renewable resources have given new impetus to interest in natural fibers as an eco-friendly alternative to conventional synthetic fibers^8,9^.

Natural fibers, despite several advantages, suffers from a few inherent disadvantages that limit the potentials of natural fibers for wide applicability in polymer composites. One of their main disadvantages is their hydrophilic nature, which results in poor interfacial adhesion with the hydrophobic nature of most of the polymer matrices. This incompatibility causes weak interfacing bonding, which results in a reduction of mechanical properties and durability of composites^10,11^. Natural fibers also tend to absorb moisture, which will cause them to swell and exhibit poor dimensional stability along with a loss of mechanical properties in a period. The natural fibers are also prone to biological degradations. All such drawbacks make natural fibers unsuitable for long-term applications under the harsh of environmental conditions. Thus, treatments for surface modification are performed to improve the performance and stability of the natural fiber-reinforced composites^12,13^.

Chemical treatments are widely used to improve natural fiber properties. From various chemical modifications, the alkali treatment is highly favourable^14^. Alkali treatment involves the soaking of fibers in a solution of sodium hydroxide (NaOH) that removes impurities from the fiber surface, such as wax, lignin, and hemicelluloses. It also cleans the fiber surface, increasing the surface roughness and improving mechanical interlocking with the polymer matrix, which in turn improves the adhesion at the interface^15^. It also breaks hydrogen bonds of the cellulose structure and hence causes fibrillation of fibers by exposing more active groups, which increases their compatibility with the matrix. It enhances the mechanical properties, reduces moisture absorption, and hence makes alkali-treated fibers more suitable for composite applications^16^.

Various benefits of the alkali treatment to improve the properties of many natural fibers have been reported by different researchers. Saha et al. reported that jute fibers, after treatment with different concentrations of NaOH solution for different immersion time, showed a 50% improvement in tensile strength for 4 wt.% of NaOH for 30 min^17^. Gomes et al. found that the 10 wt.% NaOH treatment enhanced fracture strain and toughness of composites prepared out of curaua fiber and PLA^18^ . In addition, Cai et al., concluded that a treatment with 5 wt.% NaOH for 2 h enhanced tensile strength and Young’s modulus by 8% and 36% respectively, but on the other hand increase in concentration beyond 5 wt% reduced the mechanical properties., which underlines the necessity of optimization for different alkali concentrations^12^.

While alkali treatment is very effective, other chemical methods have been examined to modify the surface properties of natural fibers such as silane coupling, acetylation, and benzoylation. Silane coupling agents form covalent bonds with both the fiber and the polymer matrix, which improves interfacial adhesion and moisture resistance^13,19^. Silane treatments are generally costlier and less environment-friendly compared to alkali treatment. One such treatment is acetylation, in which hydroxyl groups in cellulose are replaced by acetyl groups; this reduces the hydrophilicity of the fibers. However, if not adequately controlled, this may result in poor mechanical properties^20^. In the case of benzoylation, although it enhances the compatibility of the fiber and matrix, it involves dangerous chemicals and is hence less eco-friendly^21^. However, despite these processes, alkali treatment became most popular due to its simplicity and costs involved, with significant improvements obtained in the fiber properties while preserving their structural integrity.

For the optimum concentration of NaOH, treatment time, and temperature, it has been demonstrated in research that each is specific for every type of fiber. In the case of bamboo fibers, the optimal improvement of mechanical properties was obtained with a 6 wt.% NaOH treatment for 4 h^22^, whereas the treatment of abaca fibers by 5 wt.% NaOH for 2 h had produced the best result^12^. Another important factor is that the efficiency of the treatment depends on the application of the fibers. The alkali-treated hemp fibers have proven very effective in reinforcing the mechanical properties of bio-based composites used in automotive applications, while the high stiffness and thermal stability of the alkali-treated flax are more suitable for construction materials^23^. With such variability in mind, individualized alkali treatment protocols would be needed depending on specific uses to obtain an optimal improvement in fiber properties.

Raw and NaOH-treated *Tinospora cordifolia* fibers are investigated in the present work. Belonging to the scarce group of natural fibers, *Tinospora* could represent a promising reinforcing material due to its chemical composition and mechanical properties. The effectuality of NaOH treatment for property improvements in *Tinospora* fibers will be assessed to explore the possibility of employing treated *Tinospora* fibers as an environmentally friendly reinforcement of composite materials. This work is focused on how mechanical properties, surface morphology, and thermal stability of *Tinospora* fibers are influenced by different NaOH concentrations, with the aim of gaining a better understanding of the role this new fiber plays in sustainable material science. The results will add to the growing research activity related to natural fiber modifications and their applications in eco-friendly composites.

## Materials and methods

### Materials, fiber extraction and surface treatment

The tinospora cordifolia stems were obtained from localities of Manipal in Udupi district. The district experiences an average annual rainfall of about 4000 mm and a humid climate. After soaking the stems in water for 10 days, the fibers were carefully separated by hand. Later, these separated fibers were cleansed with distilled water and completely dried. Some of the extracted fibers where chemically treated with an alkaline solution, i.e. NaOH solution. The concentration of the solution chosen was 3% (w/v) and the immersion time was 90 min.

### Physical characterization

Diameter measurements were carried out by optical microscope measurements. Measurements were made on 5 fibers and atleast 5 diameter measurements were made from each fiber.

The density of the fiber was measured using the pycnometer method, with distilled water serving as the immersion liquid. The fiber density was calculated using Eq. (1), where m_1_, m_2_, m_3_ &m_4_ represent the masses (in grams) of the empty pycnometer, pycnometer filled with Tinospora cordifolia fiber, pycnometer filled with distilled water, and pycnometer filled with both natural fiber and distilled water, respectively. Additionally, ${\rho }_{w}$ denotes the density of distilled water in g/cm^3.^ This method was also employed elsewhere in literature^24^.

1 $${\rho }_{fiber}=\left(\frac{{m}_{2}-{m}_{1}}{\left({m}_{3}-{m}_{1}\right)\left({m}_{4}-{m}_{2}\right)}\right){\rho }_{w}$$

### X-ray diffraction (XRD) analysis

The X-ray diffraction test was conducted using a Rigaku Miniflex 600. It generates CuKα monochromatic radiation at 0.154 nm in wavelength. It was noted for 2θ values between 10º and 80º. Crystallinity Index (C.I) was calculated from Segal method using the height of crystalline and amorphous peaks.

Segal’s equation, i.e. Equation (2) is^25^

2 $$\text{Crystallinity Index }\left(\text{CI}\right)=\frac{{I}_{200}-{I}_{AM}}{{I}_{200}}$$

where *I*_200_ indicates the maximum intensity of 200 lattice plane at a 2θ angle corresponding to crystalline peak and *I*_*AM*_ indicates the minimum intensity between two major crystalline peaks.

Crystallite size (C.S) was calculated according to Scherrer’s Eq. ^26^, i.e. Equation (3):

3 $$\text{Crystallite Size }(\text{C}.\text{S}) =\frac{K\lambda }{\beta cos\theta }$$

K = 0.89 provides Scherrer’s constant in this case. Here, λ stands for wavelength which is equal to 0.154 nm and β is peak’s entire breadth at half maximum also called as Full Width Half Maximum (FWHM). FWHM is in radians and $\theta$ is half of the range of 2 $\theta$ under the crystalline peak in radians.

### Morphological analysis

The surface morphology of the *Tinospora cordifolia* fibers was studied using Zeiss Gemini-300 HR-FESEM with Schottky type field emitter. The fibers were gold coated prior to the microscopic examination. The areas of fibers were magnified to different magnifications to analyze the details.

### Fourier transform-infrared (FTIR) spectroscopy analysis

Using a Shimadzu spectrometer, Fourier transform infrared spectra were captured. Measurements were performed in transmittance mode throughout the 400–4000 cm^−1^ range. FTIR analysis was used to determine the functional group components found in the Tinospora cordifolia fiber.

### Thermogravimetric analysis (TGA)

PerkinElmer was used to measure the *Tinospora cordifolia* fiber’s thermal stability. Alumina crucible containing six milligrams of powdered fiber samples was put in a furnace with a regulated nitrogen flow rate of 20 ml per minute. There is a temperature range of room temperature to 800 °C. Heating rate of 10 °C each minute was maintained.

### Single fiber tensile test

In accordance with ASTM D3379, the tensile tests were carried out on Tinospora cordifolia fiber samples using a Zwick Roell machine. For a gauge length of 50 mm, testing was conducted at a crosshead speed of 8 mm/min for 20 samples. However, stress strain curves of only 5 samples are shown in this paper but average values of all 20 samples are reported for tensile strength and Young’s modulus.

## Results and discussion

### Physical characterization

**Fig. 1:**
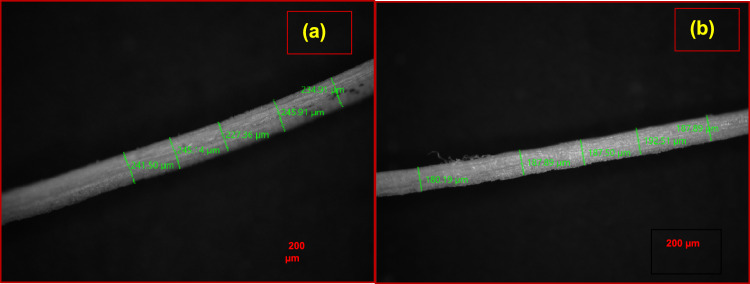
Optical microscopic images of *Tinospora cordifolia* fiber showing diameters (**a**) before treatment (**b**) after treatment.

Figure 1 shows the optical microscopic images of raw tinospora fiber and NaOH treated fiber respectively. The average value of diameters of raw tinospora cordifolia was equal to 230 ± 25 µm and after NaOH treatment it reduced to 185 ± 30 µm. The volumetric density of the raw fiber calculated from Eq. (1) was 0.6 gm/cm^3^ which increased to 0.8 gm/cm^3^ post treatment. The reduction in diameter is due to removal of hemicellulose, lignin and other impurities^27,28^. The NaOH treatment enhances the mass for a given volume of fiber, hence the increase in density^28^. It can be noted that the diameter value is in lower end of the spectrum when compared with diameter value of popular natural fibers such as coconut, bamboo and bagasse fibers^29^. Therefore, Tinospora cordifolia fiber is very light and is expected to have excellent specific properties.

### XRD analysis

**Fig. 2:**
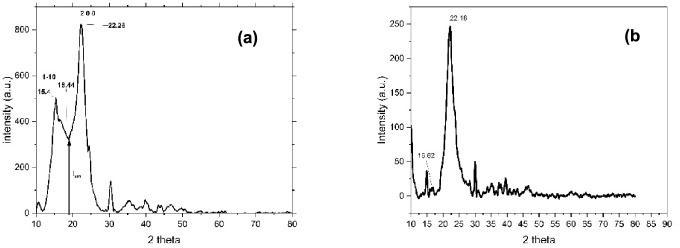
XRD curve of (**a**) raw *Tinospora cordifolia* fiber ^34^ (**b**) NaOH treated tinospora cordifolia fiber.

From the XRD analysis of raw fiber Fig. 2a, two prominent peaks were observed at the 2θ ranges of 22.26° and 15.4° in the XRD diffractograms, corresponding to the lattice planes 2 0 0 and 1 -1 0, respectively (ICDD PDF card no:00–056-1718)^30,31^. According to the ICDD card no: 00–056-1718, these peaks represent the presence of cellulose 1β^30,32^. The crystallinity index was calculated using Segal’s equation, utilizing the peak height of the 2 0 0 plane and the amorphous height measured between the minimum of the 1 − 1 0 and 2 0 0 planes. The crystallinity index was found to be 60.19%, and the crystal size was calculated to be 2.56 nm. The XRD curve after NaOH treatment is shown in Fig. 2b. The intensity of amorphous phase is the intensity corresponding to tiny broader shoulder at 16.62° and the intensity of crystalline peak is the intensity of peak at 22.16°. The calculated Crystallinity Index hence calculated from Eq. (1) is equal to 95%. The Crystallite Size (C.S) is calculated to be 3.52 nm. It can be observed that, NaOH treatment has enhanced Crystallinity Index (C.I) and Crystallite Size (C.S). The removal of amorphous contents from the fiber due to alkali treatment has led to the enhancement of C.I and narrowing of the crystalline peak.^33^ .

### Morphological analysis

**Fig. 3:**
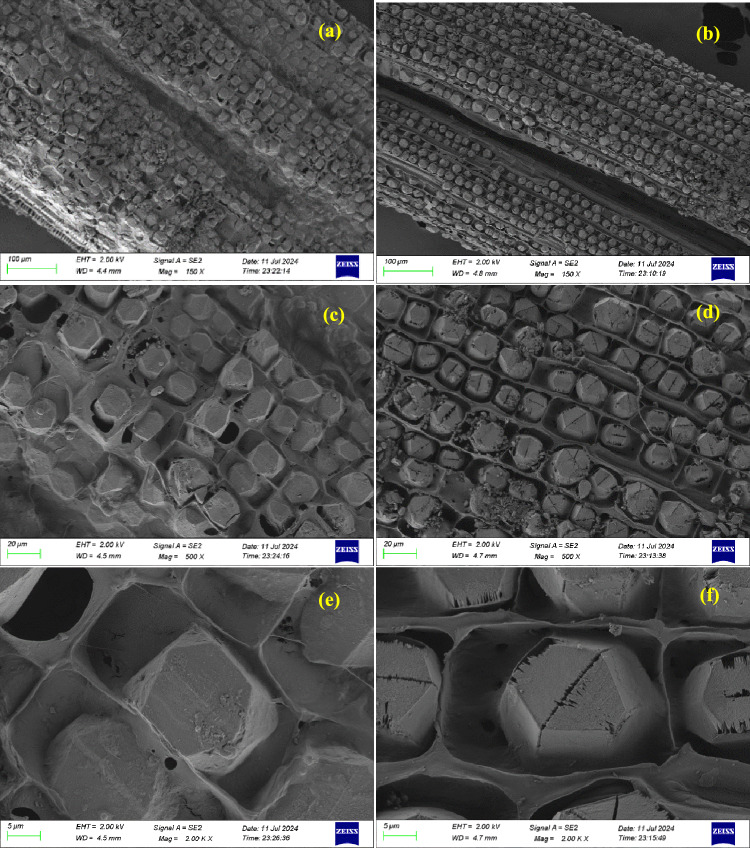
SEM images of raw (**a**, **c** & **e**) and NaOH treated (**b**, **d** & **f**) tinospora cordifolia fibers at different magnifications.

From Fig. 3a b, it can be observed that SEM images of Raw *Tinospora cordifolia* fibers appears relatively smooth because the cellulose core is enclosed in a thick outer layer that consists of lignin, hemicellulose, wax, as well as other non-cellulosic materials^15^. After alkali treatment, NaOH removes considerable amounts of lignin, hemicellulose, and wax from the surface of the fiber. As such, it exposes the cellulose portion and presents an irregular, roughened surface ^16,35^ which is evident in Fig. 3b. This roughness is important in enhancing the mechanical interlocking ability of the fiber with a polymer matrix, leading to improved adhesion. Raw fibers have non-cellulosic impurities such as wax, hemicellulose, and lignin. From Fig. 3c, it is noticed that, non-celulosic material appear as sticky patches surrounding the cellulosic core. On the other hand from Fig. 3d, it is understood that treated fibers show more surface area of the cellulose as the surrounding non-cellulosic content is removed. This can be explained by fibrillation, which is the splitting of fiber bundles into finer fibrils^28^. The barriers made of lignin and hemicellulose break, as indicated by the separated fibrils, with the opening of cellulose microfibrils hexagonal structure Fig. 3d ,f. In the untreated fibers, starch grains and other non-cellulosic content exist due to which there are fewer surface pores or voids-Fig. 3c,e. After the treatment, there are more pores and pits on the fiber surface (Fig. 3f), mainly due to the degradation of layers consisting of both hemicellulose and lignin.

### FTIR analysis

**Fig. 4:**
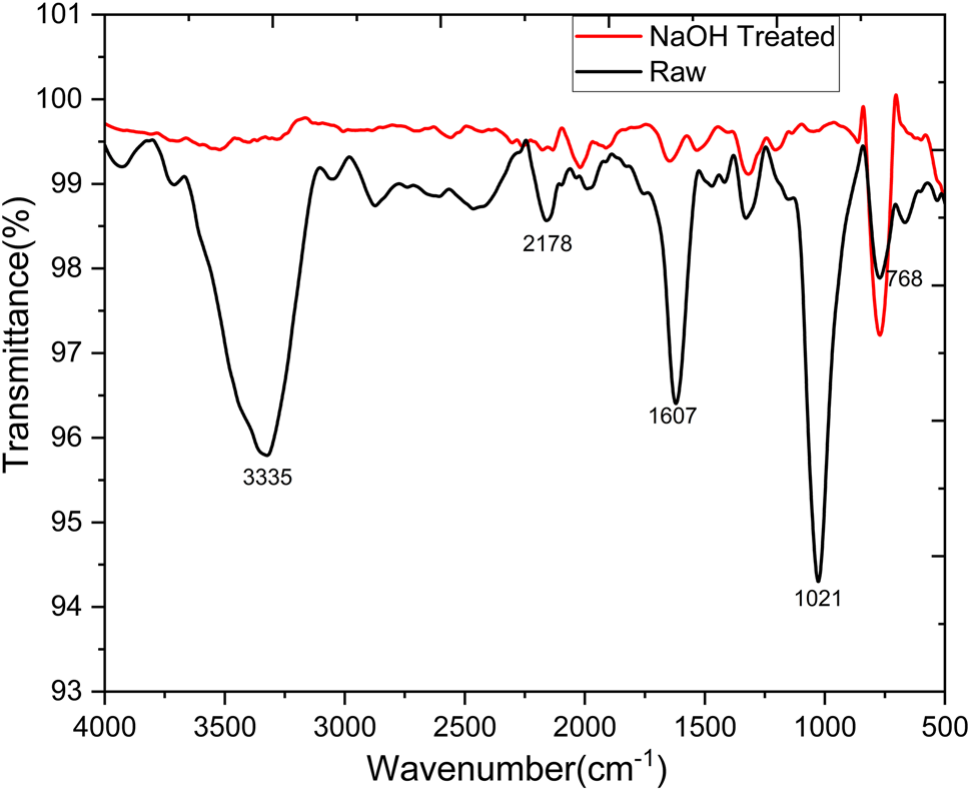
Fourier transform Infrared (FTIR) spectra of *Tinospora cordifolia* fiber raw and treated.

The FTIR spectra of raw and treated fiber is shown in Fig. 4. The peak at about 3300 cm⁻^1^ in the FTIR spectra generally represents O–H stretching vibrations, usually because of the existence of hydroxyl groups (-OH) in natural fibers^15,16,35^. Due to their hydrophilic nature, this may further implicate the absorption of moisture, which compromises the mechanical strength and other properties of composite materials reinforced with natural fibers^28^. The peak at 3335 cm⁻^1^ is removed after NaOH treatment because it takes away lignin and hemicelluloses on the surface of the fiber, in addition to removal of some hydroxyl groups which are usually attributed to cellulose and which help make the surface less hydrophilic. By reducing the content of hydroxyl groups, NaOH treatment may increase the ability of a fiber to be compatible with a hydrophobic polymer matrix, thus assisting in improving the composite interfacial bonding^36,37^. The FTIR spectra of natural fibers can show a peak at about 1700 cm⁻^1^, normally attributed to the stretching vibration of carbonyl (C = O). This kind of peak is easily traced in the existence of hemicellulose, lignin, or pectin, which are common in natural fibers and have carbonyl groups^38^. Treatment with NaOH breaks down the hemicellulose and lignin, which leads to peaks related to the carbonyl group. The decrease or disappearance of the peaks is due to the elimination of chemical bonds associated with carbonyl groups^39^. Cellulose makes no significant contribution to carbonyl stretching in this region; thus, the decreased peak at about 1700 cm⁻^1^ indicates that structural composition of the fiber has now become more amenable for composite applications^40^. There are also important C-O bonds in hemicellulose that make contributions to the peak at about 1000 cm⁻^1^. Its removal through NaOH treatment reduces the intensity of this peak. The increase in the peak around 700 cm⁻^1^ in the FTIR spectrum after NaOH treatment (alkali treatment) usually corresponds to changes in cellulose crystallinity because of the removal of amorphous materials such as hemicelluloses, lignin, and other impurities. This might contribute to the fact that it enhances the crystalline regions of cellulose; therefore, one can just deduce that, intensification of peaks can be related to an ordered structure of cellulose and in turn more crystallinity of the fiber^41–43^.

### Single fiber tensile test

**Fig. 5:**
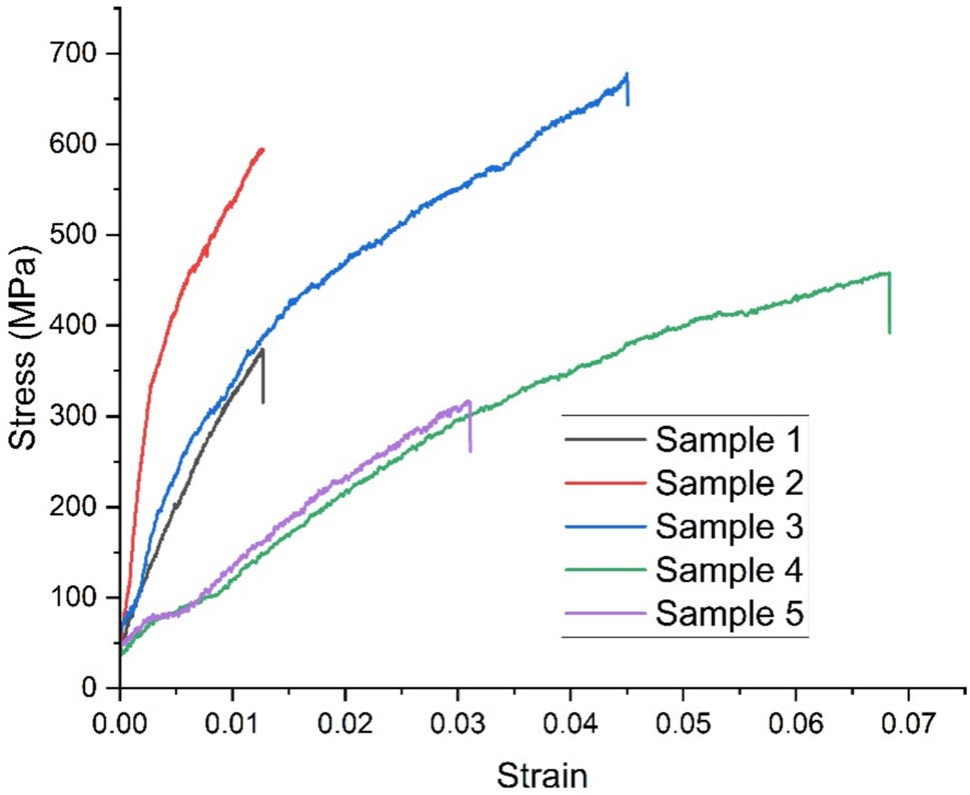
Stress–strain curves of raw *Tinospora cordifolia* single fiber ^34^.

**Fig. 6:**
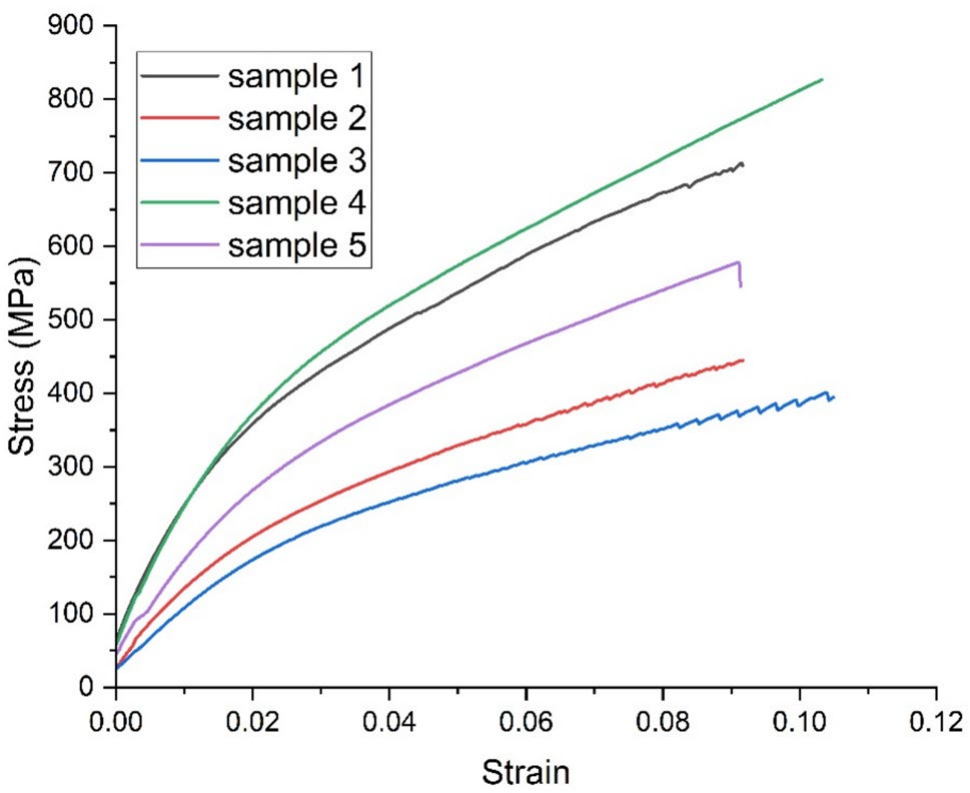
Tensile stress strain curves of NaOH treated *Tinospora cordifolia* single fiber.

**Fig. 7:**
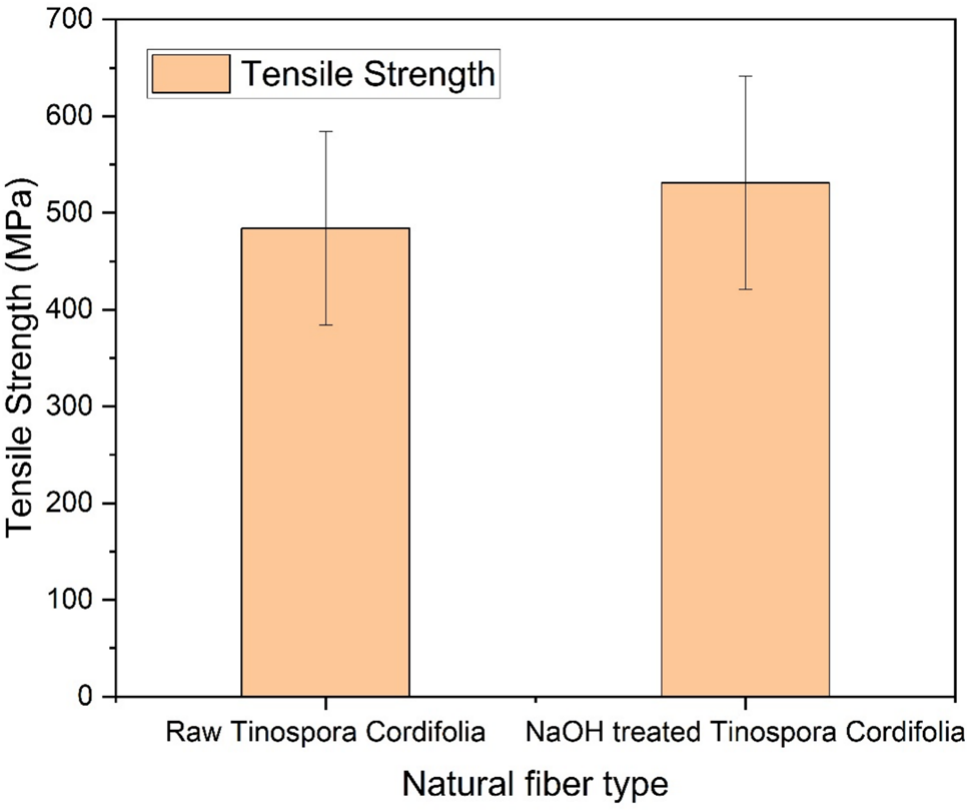
Tensile strength comparison of raw and NaOH treated *Tinospora cordifolia* fiber.

The tensile properties of natural fibers are influenced by their chemical composition (including the presence of cellulose, hemicellulose, and lignin), structure, growing conditions, harvesting time, and extraction method. Low-density fibers with enhanced tensile properties are preferred for lightweight composite structures. Figure 5 & 6 presents the stress–strain curves for five samples each of Tinospora cordifolia fiber raw and NaOH treated. Although a total of 20 samples were tested, only the graphs for five samples are shown here. Comparison of values of tensile strength, Young’s modulus and percentage elongation at break are depicted in the bar graph in Fig. 7, 8 & 9 respectively. The values of tensile strength of raw fiber are comparable with that of jute, sisal, snake grass, pineapple, flax, kenaf, ramie, cotton and hemp. It is interesting to note that the tensile strength values are higher when compared with bamboo, coir, palmyra palm and oil palm fibers^44^. The Young’s modulus values are much higher when compared with bamboo, coir, snake grass, pineapple, oil palm and cotton fibers. It is comparable with the modulus values of jute, banana, sisal, bagasse, flax, ramie and hemp. It is noticed from the bar graphs, that the tensile strength enhances by approximately 10%, Young’s modulus reduces by 30% and percentage elongation at break enhances by 148%. The reason for enhancement in tensile strength is the removal of hemicellulose, lignin, wax and other impurities leaving the well-ordered realigned cellulose chains. But at the same time, Young’s modulus reduces because of the increase in flexibility of the fiber which is evident from the increase in percentage elongation at break. This is due to removal of matrix or cementing material surrounding the microfibrils and presence of more pores and voids in the microstructure of the treated fiber, which is evident from the SEM images. Therefore, alkali treatment has resulted in a fiber that is stronger and more flexible.

**Fig. 8:**
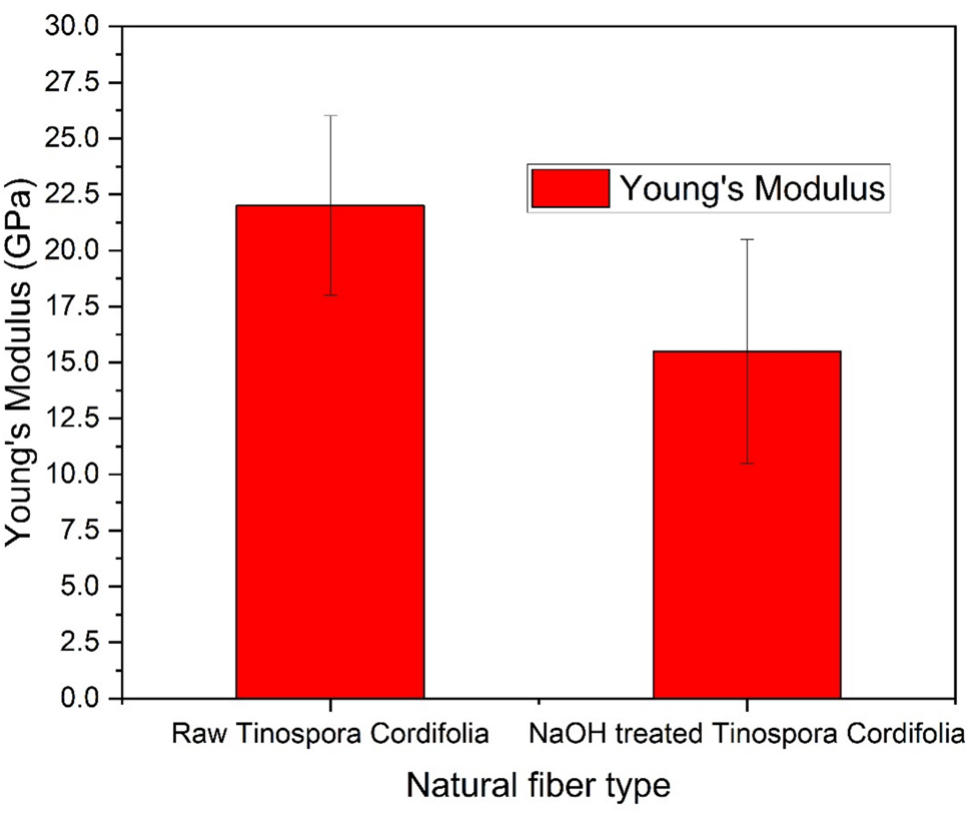
Young’s modulus comparison of raw and NaOH treated *Tinospora cordifolia* fiber.

**Fig. 9:**
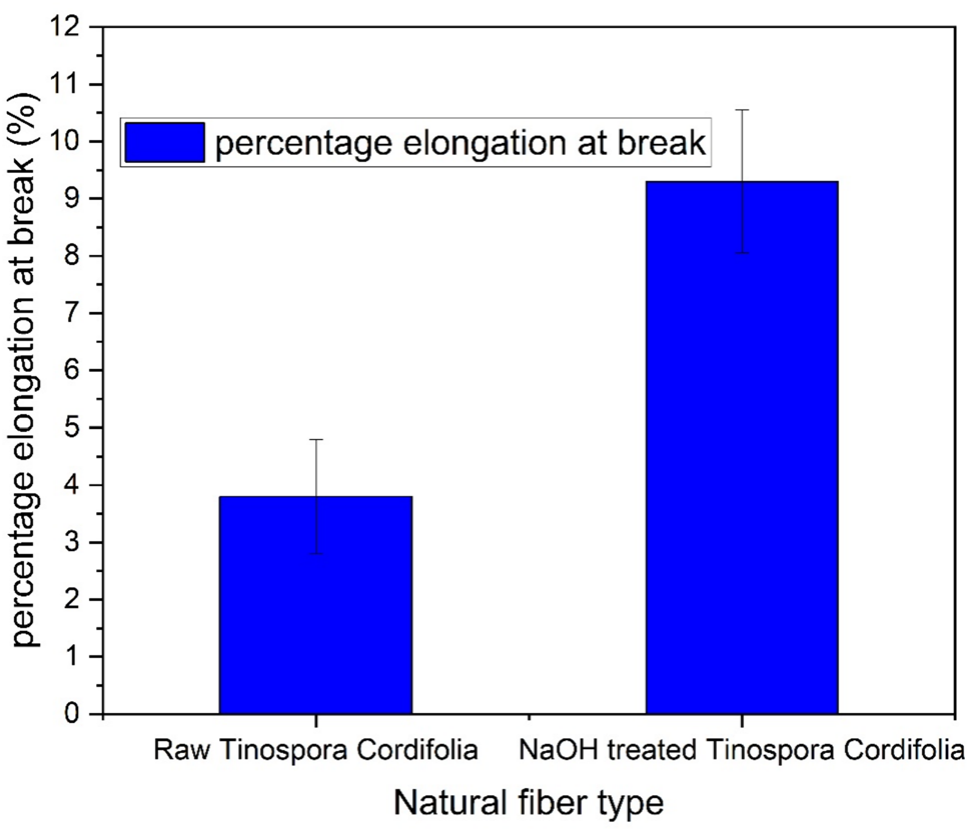
Percentage elongation at break comparison of raw and NaOH treated *Tinospora cordifolia* fiber.

### TGA

**Fig. 10:**
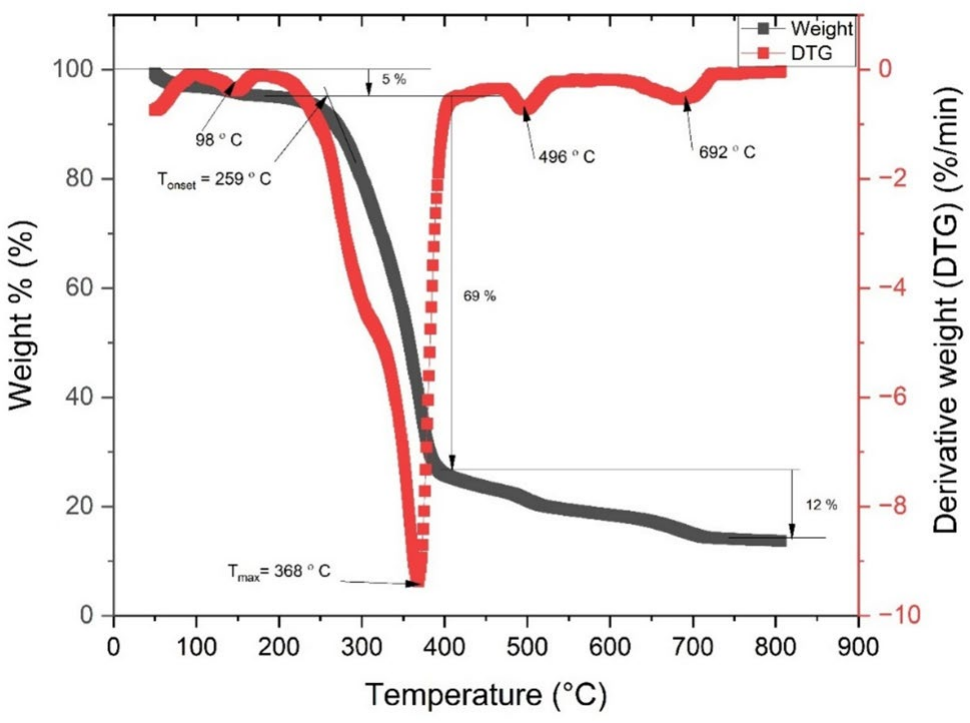
Weight loss (%) and derivative weight loss (%/min) as a function of temperature of raw fiber^34^.

**Fig. 11:**
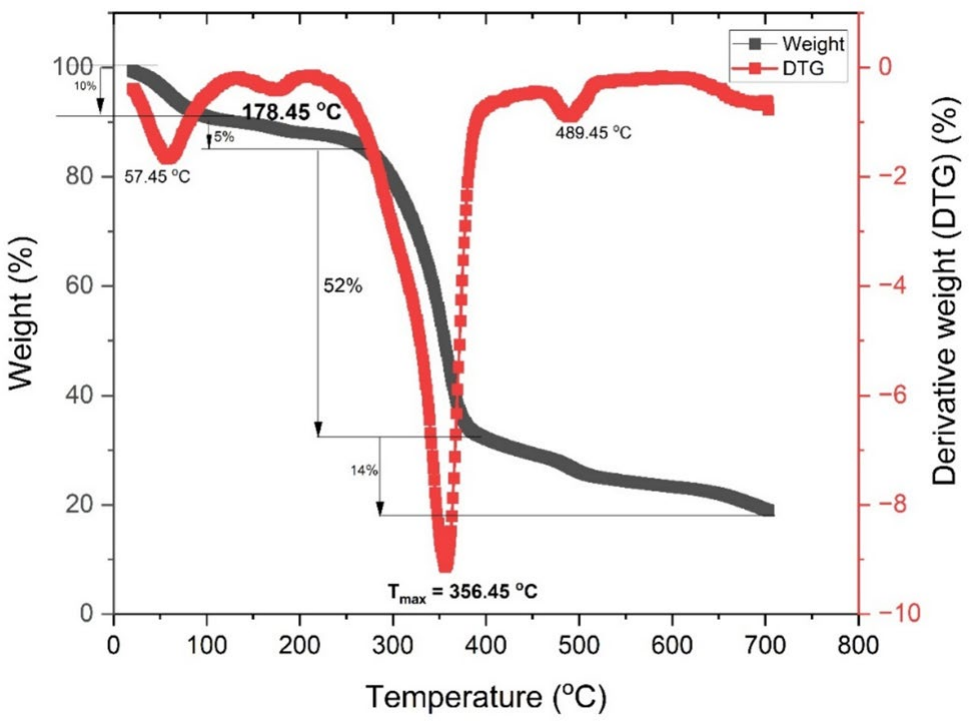
Weight loss (%) and derivative weight loss (%/min) as a function of temperature of NaOH treated fiber.

Figure 10 & 11 shows the weight loss and derivative weight loss curves as a function of temperature for raw and NaOH treated fiber respectively. In the raw fiber, the degradation of Tinospora cordifolia occurs in clear three stages – first stage from room temperature to 169 °C, second stage from 169 to 397 °C and third stage occurs from 397 to 716 °C. In the first stage, evaporation of moisture peaks at 98 °C and 5% mass loss is observed^45^ . In the second stage overall mass loss is 69%, which includes mass loss of hemicellulose and cellulose ^46^. The mass loss of hemicellulose constituent is observed as a shoulder around 300 °C. The mass loss of cellulose peaks at 368 °C, which is considered as maximum thermal degradation temperature, T_max_. The onset of thermal degradation, T_onset_ is observed at 259 °C In the third phase mass loss of lignin and wax is observed which is evident from peaks at 496 and 692 °C^47,48^.

In NaOH treated fiber, 4 stages of thermal degradations are identified. The comparison of different stages is shown in Table 1. From Table 1, it is noted that the thermal stability usually denoted by T_onset_ has enhanced post NaOH treatment. However, the maximum degradation temperature T_max_ reduced slightly.

**Table 1:** Comparison of stages of degradation/ thermal stability of raw and NaOH treated *Tinospora cordifolia* fiber.

		Raw	Treated
T_onset_ (˚C)		259	280
T_max_ (˚C)		368	356.45
1st stage of degradation	Temp range(˚C)	50—259	10–130
	Weight loss (%)	5	10
	Peak Temp(˚C)	98	57.45
2nd stage of degradation	temp range(˚C)	259–400	130–280
	weight loss (%)	368	5
	Peak Temp(˚C)	98	178.45
3rd stage of degradation	Temp range(˚C)	400–800	280–392
	Weight loss (%)	14	52
	Peak Temp(˚C)	496 & 692	356.45
4th stage of degradation	Temp range(˚C)	NA	392–710
	Weight loss (%)	NA	14%
	Peak Temp(˚C)	NA	489
Residual mass	%	12	19

Stage 1 degradation which is usually below 100 °C is due to evaporation of water molecules. The next two stages are due to degradation of pectin, hemicellulose, lignin and due to the cleavage of glycosidic linkages of cellulose. The final stage is usually due to degradation of lignin and alpha cellulose^49,50^.

T_onset_ of NaOH treated fiber is greater than that of popular natural fibers namely kenaf, hemp, sisal, coil and pineapple leaf fibers^51^.

### Comparison of properties of common natural fibers with *Tinospora cordifolia*

In the Table 2, NaOH treated Tinospora Cordifolia, and some common natural fibers are compared. NaOH treatment parameters are different for different natural fibers. Properties such as tensile strength, Young’s modulus, maximum degradation temperature and Crystallinity Index are populated in the table against each fiber and its respective treatments. The tensile strength of treated Tinospora Cordifolia is comparable with that of treated jute and flax. Maximum degradation temperature of the fiber under study is close to that of bamboo and flax fibers. The reason for enhancement in tensile and thermal properties is due to increase in Crystallinity Index by 50%. NaOH treatment has effectively removed the amorphous phase in the natural fiber which is clear from the study. The treatment is successful in enhancing the properties of fiber and making it competitive with natural fibers such as jute, flax and bamboo. Literature review on NaOH treatment of different natural fibers revealed the fact that NaOH treatment is effective especially at concentration of 5 wt.% or 5% (w/v) improving the crystallinity of natural fibers which in turn enhances tensile strength and thermal stability of the fibers. NaOH concentration play a vital role which can be observed from the effect of varying concentration on the properties of bamboo, coir & pineapple fibers. As the concentration increases crystallinity and tensile properties enhances only upto 5%, beyond which because of cellulose chain scission, dip in values of these parameters is observed^52^. From the review on coir, pineapple & flax fibers, it is observed that NaOH immersion time has also significant effect on the properties. In case of flax fibers, it can be noticed that there is an optimum immersion time beyond which fiber properties decrease. It is at this time that the stress transfer between the microfibrils is optimum^53^.However, this optimum immersion time depends on concentration, fiber type and immersion temperature. This can be strikingly observed in the case of flax fiber when treated at much higher temperature. At higher temperature, the optimum immersion time is much lower^54^.

**Table 2:** Comparison of properties of common natural fibers with* Tinospora cordifolia* in their treated state.

Natural fiber	NaOH treatment parameters	Optimum treatment conditions	Tensile strength (MPa)	Tensile modulus (GPa)	Max. degradation temperature (˚C)	Crystallinity Index (%)	Refs
*Tinospora cordifolia* NaOH treated (current study)	**3% (w/v), 90 min, RT**	**NA**	**531 ± 110**	**15.5 ± 5**	**356.45**	**90**	**NA**
Jute	1–6 wt.%, 30 min, 30 °C	5 wt.%	610	38	316.3	72	^55^
Banana	5 wt.%, 24 h, RT	5 wt.%	189.7	-	340	47.62	^56^
Bamboo	5 wt.%, 24 h, RT	5 wt.%	-	-	353.26	69.25	^57^
	1 wt.%, 3 wt.%, 5 wt.% & 7 wt.% at RT, 24 h	5 wt.%	119.17	^-^	-	76.87	^58^
	5%(w/v), 60–65 °C, 1 h	5% (w/v)	225	12.2	-	-	^59^
Coir	5%,10%,15%,20%	5% for crystallinity, 20% for tensile strength	348	-	-	62.33 for 5%	^60^
	Full factorial DOE—3 factors 3 levels (concentration—1, 5, 20%, temp.—30, 50& 80 °C & duration—24, 48 & 72 h)	20%, 50 °C, 24 h	250	3.2	-	-	^61^
Pineapple	1,3,6, 9 wt.%, 1 h, 26 °C	6% for tensile strength and modulus	1620	27	-	75.6	^52^
	4, 6 & 8% (w/v), 1 & 3 h	6% for 1 h Thermal stability, 6% for 3 h for tensile strength	164.55	-	308.34	-	^62^
Flax	5% & 20% (w/v), 30 min, RT	5%	-	-	366	76	^63^
	2% & 5%, 30, 60 & 90 min	2%, 60 min	760.21	-	380.46	-	^53^
	2%, 80 °C, 30 min, 1 h, 2 h & 4 h	2%, 80 °C, 30 min for tensile properties, 2%, 80 °C & 2 h for thermal stability	569.2	35	361.3	-	^54^

## Conclusions

The study presents a comprehensive analysis of the effects of NaOH treatment on the properties of Tinospora cordifolia fibers and discusses the potential of these fibers as suitable natural reinforcements for sustainable composite materials. The decrease in fiber diameter from 230 µm to 185 µm with an increase in density from 0.6 to 0.8 g/cm^3^ was due to the removal of hemicellulose, lignin, wax, and other impurities as indicated by XRD analysis. Fiber crystallinity index (C.I.) increased significantly after treating the fiber, owing to the efficient removal of non-cellulosic components, which was further confirmed with SEM images that also revealed increased surface area due to extensive fibrillation, the formation of clear hexagonal structures, pores, and voids. NaOH treatment improved tensile strength by about 10%, whereas the elongation at break increased by 148%, and Young’s modulus decreased by 30% due to the better ductility. Thermogravimetric analysis demonstrated different stages of degradation as well as an 8% increase in thermal stability, indicating that the fiber showed a lower susceptibility for thermal degradation. Higher crystallinity of cellulose was obtained because its amorphous material was removed and the freshly created finer micro-fibrillation enhanced mechanical properties and thermal characteristics of treated fibers. These changes did have an effect on density, but the modified fibres remained light and consequently suitable for lightweight applications.

These results confirm the emerging class of natural fiber composites by showcasing, for the first time, Tinospora cordifolia as a potential substitute to synthetic reinforcements. Further studies are needed to develop optimal surface treatments to improve fiber-matrix bonding, investigate hybrid composite formulations, and evaluate long-term durability under a range of environmental conditions. The study provides a strong foundation for further advancements in sustainable composite materials, paving the way for eco-friendly engineering applications.

## Data Availability

The datasets used and/or analysed during the current study are available from the corresponding author on reasonable request.

## Declarations

### Identification statement

The plant was identified by G K Ramesh Rao, Lecturer in Botany, Mother Teresa’s PU College, Kundapur Taluk, Udupi, Karnataka, India. There are couple of other published articles on morphological & mechanical characteristics of the natural fiber and several studies on medicinal properties of the same. Our work is on physical, morphological & thermal characteristics applicable to composite materials. The fiber or the plant is not stored in any public herbarium. The plant is widely available locally and not in the danger of extinction.
